# Factors associated with potential doping behavior among Swedish combat sport athletes: a cross-sectional study

**DOI:** 10.3389/fspor.2026.1954492

**Published:** 2026-10-07

**Authors:** Jonathan Karl Oliver Myléus, José Morales, Haris Pojskic, Damir Sekulic

**Affiliations:** 1 FPCEE, Blanquerna Ramon Llull University, Barcelona, Spain; 2 Department of Sports Science, Linnaeus University, Kalmar, Sweden; 3 Faculty of Kinesiology, University of Split, Split, Croatia; 4 High Performance Sport Center, Croatian Olympic Committee, Zagreb, Croatia

**Keywords:** alcohol misuse, competitive fighting, dietary supplements, doping likelihood, martial arts, tobacco, training load

## Abstract

**Introduction:**

The global growth and commercialism in combat sports have resulted in practitioners using performance-enhancing substances such as androgenic anabolic steroids, diuretics, hormone modulators, or stimulants. To gain a competitive edge, pressure often makes experienced athletes more prone to doping. Despite global trends of doping in combat sports, there is an evident lack of knowledge on protective and risk factors related to doping behavior in combat sports athletes. This study aimed to investigate (i) the prevalence of potential doping behavior (PDB) in Swedish combat sport athletes, (ii) sexbased differences in PDB, and (iii) factors associated with PDB.

**Methods:**

A crosssectional observational study was conducted using a digital survey approach. The study population consisted of 120 active male (age: 40.6 ± 13.0 years) and 45 female members (age: 40.6 ± 9.2 years) of combat sport clubs in Sweden. Two standardized questionnaires were used: (1) Knowledge of Doping Questionnaire (KD), and (2) Questionnaire on Substance Use (QSU). The data were analyzed using Binomial Logistic Regression analysis and Fisher's Exact Test.

**Results:**

The prevalence of PDB was 9.7%. The logistic regression analysis showed “Training load” (OR = 1.005) and “Occasional tobacco use” (OR = 7.178) were positively associated with PDB. In other words, occasional tobacco users had over seven times higher odds of PDB compared to non-users. Also, each unit increase in training load slightly elevated the risk for PDB. Additionally, sexbased differences showed males as more prone to PDB compared to their female counterparts (*p* = 0.007). All other covariates, including “Doping knowledge*,”* “Daily tobacco use*,”* “Trust in coach expertise in doping*”* and “Competitive results*”* were not significantly associated with PDB.

**Discussion:**

In conclusion, for Swedish combat sport athletes, PDB was associated with higher training load and occasional tobacco use. Consequently, future antidoping interventions should address these identified associations in doping prevention programs.

## 1 Introduction

The use of prohibited performance-enhancing substances remains one of the major challenges to modern sport, threatening athletes' health, the integrity of competition, and the fundamental principles of fair play (1, 2). Although anti-doping programs have substantially evolved over recent decades through advances in testing procedures, biological monitoring, and educational initiatives, doping continues to be reported across a wide range of sporting disciplines (3). Consequently, the global fight against doping is highly prioritized within the sport community.

Rather than focusing exclusively on confirmed anti-doping rule violations, contemporary research has emphasized the importance of identifying athletes who may be more susceptible to future doping behavior (4, 5). In this context, the concept of doping intentions (potential doping behavior—PDB) has emerged as a valuable research construct, enabling the identification of protective and risk factors associated with doping susceptibility before actual doping occurs (6–9). Henceforth, PDB is used as the operational term for CS athletes' doping intentions or susceptibility to future doping. Thus, in this manuscript, “doping intentions” and “PDB” refer to the same underlying construct of self-reported willingness or openness to future doping, rather than confirmed doping behavior. Such evidence provides an opportunity to recognize vulnerable athlete populations and to develop targeted, sport-specific prevention strategies that address the underlying determinants of doping behavior rather than its consequences alone.

Although numerous studies have investigated correlates of PDB, their findings have not been entirely consistent. Factors such as sex, competitive achievement, doping-related knowledge, substance use, nutritional practices, and coaching characteristics have all been identified as potential determinants of doping susceptibility (7, 8, 10). However, the direction and magnitude of these associations appear to depend largely on the specific sporting context. Also, previous research and WADA reports have demonstrated considerable differences in both the prevalence of doping and PDB, but also in risk factors across sports characterized by distinct physiological demands, competitive structures, and sociocultural environments (3, 11). Consequently, findings obtained in one sport cannot be readily generalized to another, highlighting the importance of sport-specific investigations aimed at identifying the determinants of doping susceptibility within specific sports.

Specifically, using comparable assessment approaches, negative doping tendencies were reported more frequently among sailing athletes, tennis players, and youth swimmers, whereas markedly greater doping susceptibility was observed in physically demanding sports such as rugby, weightlifting, and kickboxing (7, 8, 11–14). In high-level kickboxers, only 46% of participants expressed an unequivocally negative tendency toward future doping, indicating considerably greater susceptibility than that generally reported in several other sports (8). Importantly, the predictors identified in kickboxing also differed from those reported in other sports. Greater training experience and the perception that doping was prevalent in kickboxing were associated with higher PDB, whereas better knowledge of sports nutrition was protective. In contrast, competitive achievement, doping knowledge, and motivational orientation were not significantly associated with PDB (8). This differs from findings in swimming, rugby, sailing, and male team sports, where greater competitive achievement was generally associated with lower doping susceptibility (7, 10, 13, 15).

Indeed, combat sports may represent a particularly distinctive context for studying PDB because of several characteristics that differentiate them from many other sporting disciplines. Unlike the majority of individual sports (i.e., swimming, athletics, artistic gymnastics), success in combat sports is determined through direct one-on-one competition, where athletes must outperform a single opponent rather than achieve an objectively measured performance standard. Such a competitive structure may intensify the perceived importance of gaining even small performance advantages. Such pressure may be directly relevant in one-on-one competitive settings, where social comparison processes are more intense and even small performance differences have a greater psychological importance. Research on social comparison and competition suggests that motivation in competitive environments tends to be stronger with a smaller number of competitors and focus on a specific rival rather than a broad field (16, 17). In addition, combat sports are characterized by a unique sociocultural environment in which psychological toughness, tolerance to pain, and acceptance of physical confrontation are integral components of athletic identity (18). Finally, many combat sport disciplines require athletes to repeatedly engage in rapid weight loss (weight cutting), exposing them to substantial physiological and psychological stress before competition (1, 19). Collectively, these sport-specific characteristics may contribute to a pattern of doping susceptibility that differs from that observed in many other sports. This altogether clearly supports the need for dedicated investigations in combat sport populations.

Despite increasing interest in doping in combat sports, the available scientific evidence remains surprisingly limited (1). The recent systematic review dedicated to this topic identified only several eligible original studies, highlighting the scarcity of research examining doping-related behaviors in combat sport athletes (1). Existing evidence suggests that doping in combat sports is shaped by a combination of physiological, psychological, and sociocultural factors. In particular, rapid weight loss (weight cutting) has been recognized as a widespread practice across many combat sport disciplines, exposing athletes to substantial physiological and psychological stress while potentially increasing vulnerability to unhealthy and risky performance-enhancing behaviors (20).

Furthermore, psychosocial factors, including perceived social norms, self-regulatory efficacy, and the acceptance of doping within the sporting environment, have also been identified as important correlates of doping-related attitudes and behaviors among combat sport athletes (21). Consistent with these observations, already cited study in international-level kickboxers demonstrated that athletes who perceived doping as more prevalent in their sport and those with greater training experience reported higher susceptibility to potential doping behavior, whereas better sports nutrition knowledge appeared to exert a protective effect (8). However, it seems that current knowledge is largely based on heterogeneous populations and study designs, making it difficult to identify consistent determinants of doping-related behaviors across combat sports (1).

When it comes to anti-doping efforts, another issue deserves attention. Namely, although anti-doping regulations are largely harmonized under the World Anti-Doping Code, their (legal) implementation occurs within national sport systems that differ in organizational structure, educational programs, testing strategies, and cultural attitudes toward doping (9). Consequently, factors associated with potential doping behavior may vary not only across sports but also across countries. Identifying country-specific correlates of PDB is therefore essential for developing targeted prevention programs that reflect the characteristics of the local sporting environment rather than relying solely on evidence obtained from other populations. Sweden represents a relevant environment for this research because the anti-doping system has traditionally combined a public health-oriented approach, early legal criminalization of doping use, and broad prevention efforts stretching beyond elite sports (22–24). In addition, combat sports form a different sport-environment characterized by rapid weight-loss practices, competitive and social pressures, and potentially uneven anti-doping exposure across different disciplines. Thus, making Swedish CS athletes a particularly informative population in which to study potential doping behavior.

From the previous literature overview, it is evident that potential doping behavior is influenced by a complex interplay of sport-specific, individual, and sociocultural factors (1, 6, 8). Previous studies have consistently demonstrated that both the prevalence and determinants of PDB vary across sporting disciplines, while the unique characteristics of combat sports and differences between national sporting environments further limit the generalizability of existing findings (1, 15). Although several studies have examined PDB in athletes from different sports and countries, evidence specifically addressing combat sport athletes within the Swedish sporting system is currently lacking. Such evidence is needed to identify context-specific correlates of PDB and to support the development of evidence-based anti-doping prevention strategies tailored to the national sporting environment. Therefore, the aim of this study was to investigate the factors associated with potential doping behavior among Swedish combat sport athletes. Based on previous evidence, we hypothesized that sport-related characteristics, substance use, anti-doping knowledge, and training-related factors would be significantly associated with PDB.

## 2 Material and methods

### 2.1 Participants

The study population consisted of 120 active male members (age: 40.6 ± 13.0 years) and 45 active female members (age: 40.6 ± 9.2 years) of combat sport clubs in Sweden. Participants were recruited through the official registry of the Swedish Budo & Combat Sport Federation. To be included in the study, participants had to be at least 18 years of age and active athletes officially registered with the Federation. Most participants (males: 66.7%, females: 91.1%) reported university-level education. Initially, 168 responses were recorded. One participant was excluded for failing to meet the inclusion criteria. Two participants identified as “Other” gender were also excluded from the final analysis to allow for binary sex-based comparisons. The anthropometric characteristics for male and female athletes are presented in Table 1.

**Table 1:** Age and anthropometric characteristics of Swedish male and female combat athletes.

Variables	Group	N	Missing	Mean	SD	Median	Min	Max	Mann–Whitney U
Age [years]	Male	119	1	40.8	12.9	41	19	77	*p* = 0.076
	Female	45	0	40.6	9.2	41	24	62	
Height [cm]	Male	118	1	180.4	7.2	181	165	199	*p* < 0.001
	Female	45	0	167.2	6.4	168	152	180	
Weight [kg]	Male	119	1	85.4	14.8	83	57	150	*p* < 0.001
	Female	45	0	67.8	11.8	67	50	120	

N, number of participants; SD, standard deviation; Min, minimum; Max, maximum. The anthropometric variables were self-reported. Statistical significance is set at *p* < 0.05.

All participants received clear information on the study, and they provided consent that they had received, read, and understood the study's aim, the benefits, the risks, the ethical issues, and that they could terminate their participation in the study at any time. Given the sensitive nature of the research, data were coded and archived in accordance with the EU General Data Protection Regulation (2018:218). The research collected no personal information, such as date of birth, ethnicity, city of residence, or occupation. The study was approved by the Swedish Ethical Review Authority (No. 2023-0086-01) and conducted in compliance with the Declaration of Helsinki.

### 2.2 Study design

A cross-sectional observational study design was applied, using a digital survey approach. The design was structured in accordance with STROBE guidelines for observational research (25). Two standardized and validated questionnaires were used: the Questionnaire on Substance Use (QSU), which included Substance Use and Misuse (SU&M) and sociodemographic factors (26), and the Knowledge on Doping (KD) questionnaire (7). The QSU and KD questionnaires were selected *a priori*, as these standardized instruments have been previously used in research on PDB, allowing comparisons with earlier research. However, we acknowledge that these measurements only measure fractions of the complex problem discussed in the Introduction. Although the broader literature identifies psychosocial constructs such as social norms, self-regulatory efficacy, attitudes, morality, and related well-being factors as important correlates of doping intensions, these domains were not directly assessed in the present study. Therefore, the findings should be interpreted as evidence on selected correlates of PDB rather than a comprehensive test-battery of the full theoretical model of doping susceptibility.

#### 2.2.1 Independent variables

*QSU* was used to assess substance use and register sociodemographic factors such as *age, sex,* and *educational level* (26). In addition, the *SU&M* included questions about tobacco use. They consisted of “multiple choice” questions and were graded on an ordinal, Likert-based scale from one to four (8). Moreover, the highest competitive results achieved by participants (ranging from *non-competitors* to *internationally ranked medalists*) were assessed using a five-point scale. However, during analysis, the low cell count in the highest competitive-result categories caused model instability (i.e., quasi-complete separation). Therefore, the variables were recoded into a three-level categorical predictor: *Recreational/National Participation* as the reference group (including non-competitors and participation at the national level without medaling). *National Medalist* (intermediate level). *International Medalist* (Elite level with combined international medalists and top-ranked international athletes). Likewise, the initial modeling showed that including “educational level” caused model instability. The small number of participants and zero-cell counts in certain educational categories (e.g., elementary) caused exclusion from the logistic regression analysis.

The *KD* questionnaire was used to assess knowledge of banned substances and doping practices established and adopted by WADA. Several questions about erythropoietin and doping side effects were also added. Questions were formulated in statements such as “The use of amphetamines by women is related to male-like changes in body appearance.” The total score ranged from 0 to 18, representing the number of correct answers. Each question was a binary item (True/False) and was converted into a score with Correct = 1 and Incorrect = 0. The composite score of all 18 questions represented the participants' overall knowledge of doping (7).

To quantify “Training Load*,”* the total duration of weekly training was assessed by asking participants, “How much time do you train weekly?”. Response options were in a multiple-choice format and ranged from ≤4 h to ≥12 h. To estimate training intensity, participants reported how many times per week they engaged in sessions of six different perceived intensities, ranging from very easy to extremely hard. For each intensity level, responses were in a “multiple choice” format and ranged from 1 = 0 to 6 = ≥6 sessions per week. Training load was estimated as a composite internal-load score derived from weekly training duration and self-reported session-intensity frequency. Weekly duration categories were converted to numeric values as follows: 1 = ≤ 4 h, 2 = 4–6 h, 3 = 7–8 h, 4 = 9–10 h, 5 = 11–12 h, and 6 = ≥ 12 h. Perceived intensity was reported across six response levels: very easy, easy, somewhat hard, hard, very hard, and extremely hard. These columns were collapsed into three broader categories used in the formula as follows: easy = very easy + easy; medium = somewhat hard + hard, and hard = very hard + extremely hard. The grouped intensity categories were weighted as easy = 1, medium = 2, and hard = 3. The final training-load score was calculated as: TL = D × [(1 × Intensity_Easy) + (2 × Intensity_Medium) + (3 × Intensity_Hard)]. Because the metric represents a derived composite rather than directly observed session-RPE, it should be interpreted in arbitrary units.

#### 2.2.2 Dependent variable

PDB and doping-related factors were included in *QSU*, along with questions about trust in their coaches' knowledge of doping. Questions were formulated as “Do you trust your COACH regarding their advice, knowledge, and expertise in doping?”. The answer options were provided in statement format and categorized into dichotomous outcomes (i.e., “Yes/No”). In the final analysis, the dependent variable (PDB) was categorized into binary outcomes: “low risk” (0) and “high risk” (1). The binomial criterion was based on the following statement: I would grade my potential doping behavior as follows: 0 = I would not use doping; 1 = Unsure, I would use doping if there was no health risk, and I would use doping if it helped me.

### 2.3 Statistical analysis

Descriptive statistics, presented as frequencies and percentages, were calculated for all variables. Between-group comparisons for continuous variables (e.g., age, height, weight, training load, doping knowledge) were performed using the Mann–Whitney *U*-test, given the lack of normality in distributions and unequal group sizes by sex. The denominator for each analysis reflects the number of participants with available data for the specific variable. Therefore, N varies slightly across tables because of limited item-level missingness. Before the multivariate modeling, a preliminary analysis included bivariate associations between categorical predictors and the dependent variable. *Pearson's Chi-Square (x2)* and *Fisher's Exact Test* provided probability estimates for cases with low expected cell counts (<5). The Haldane-Anscombe correction was applied for this calculation due to the presence of a zero cell in the contingency table. However, since the variable “Sex-based differences” showed perfect separation (no females were classified as “Higher risk”), it was excluded from the Binomial Logistic Regression analysis to ensure model stability. A Binomial Logistic Regression was conducted for the independent variables for PDB. The final model included predictors: “Training load,” “Doping knowledge,” “Trust in coaches' expertise in doping,” “Tobacco use,” and “Competitive results”. All variables were based on theoretical relevance and statistical stability. The model evaluation included multicollinearity and Variance Inflation Factors (VIFs), with values below 2.0 considered acceptable. Nagelkerke's R2 was used for model fit and the proportion of variance explained by the model. The results were reported as unstandardized coefficients (B), Odds Ratios (OR), and 95% Confidence Intervals (95% CI). Statistical significance was set at *p* < 0.05. The primary aim of the logistic regression was explanatory and to identify factors associated with high PDB; rather than to develop a clinical prediction tool. Therefore, the analysis focuses on odds ratios and confidence intervals rather than classification thresholds or predictive performance metrics. Because the number of high-PDB cases was limited and some factors showed separation or quasi-complete separation, the regression estimates should be interpreted cautiously as exploratory associational estimates. Statistical analyses were conducted using Jamovi version 2.6.44.

## 3 Results

A total of *n* = 165 participants were included in the final analysis, representing a broad range of combat sport disciplines. The largest groups were Brazilian jiu-jitsu (BJJ; *n* = 55, 33.3%) and traditional martial arts (*n* = 52, 31.5%). The remaining participants practiced jujutsu (*n* = 11, 6.7%), other combat sports such as Krav Maga and HEMA (*n* = 10, 6.1%), Muay Thai (*n* = 9, 5.5%), kickboxing (*n* = 8, 4.8%), mixed martial arts (MMA; *n* = 7, 4.2%), wrestling (*n* = 7, 4.2%), all-style martial arts (*n* = 4, 2.4%), and boxing (*n* = 2, 1.2%). In total, the sample comprised 165 participants, with BJJ and traditional martial arts practitioners together accounting for 64.8% of the study sample.

The calculated prevalence of PDB was 9.7%. The Binomial Logistic Regression model had a deviance of 88.8 and was statistically reliable and stable (VIF < 1.10 for all predictors). In addition, the shortage of female participants in the “high-risk” group (perfect separation) led to the exclusion of the variable “Sex-based differences” from the multivariate analysis.

### 3.1 Factors associated with potential doping behavior

Because the study was designed to identify explanatory associations rather than to develop a prediction model, model performance is reported in terms of effect estimates and confidence intervals rather than sensitivity, specificity, or classification thresholds.

“Training load” were positively associated with PDB with an OR of 1.005 (Estimate = 0.00510, SE = 0.00240, Z = 2.1284, *p* = 0.033) (95% CI [1.0004, 1.01]). Practitioners using “Occasional tobacco” compared to non-users had significantly higher odds of PDB with an OR of 7.178 (Estimate = 1.97105, SE = 0.84971, Z = 2.3197, *p* = 0.02) (95% CI 1.3575, 37.96). Among low-risk PDB participants, 115 (77.2%) reported no tobacco/nicotine use, 10 (6.7%) had quit, 6 (4.0%) reported occasional use, and 18 (12.1%) reported daily use. Among high-risk PDB participants, 9 (56.3%) reported no use, 2 (12.5%) had quit, 3 (18.8%) reported occasional use, and 2 (12.5%) reported daily use. All other covariates, including “Doping knowledge,” “Daily tobacco use,” “Trust in coach” and “Competitive results”, were not statistically significant (*p* > 0.05).

The relationship between the variables “Sex-based differences” and PDB was examined with a contingency table. The OR male-to-female was 14.37 [95% CI (0.86,240.15)]. The table included 120 males and 45 females. 104 male participants were categorized as “low risk” for PDB. 16 male participants were categorized as “high risk” for PDB. All 45 female participants were categorized as “low risk” with 0 in the “high risk” category. A total of 149 participants were “low risk”, and 16 participants were “high risk” for PDB. All 16 high-PDB cases occurred among males (*n* = 120), with zero cases among females (*n* = 45). Fisher's exact test evaluated the association between “Sex-based differences” and PDB. The test demonstrated a statistically significant association (*p* = 0.007). The sex-based differences in training load and doping knowledge are summarized in Table 2.

**Table 2:** Sex-based differences in training load and doping knowledge.

Variable	Group	Missing	N	Mean	SD	Mann–Whitney U
Training load	Male	0	120	90.6	103	***p*** = 0.778
	Female	0	45	79.1	74.0	
Doping Knowledge	Male	1	119	13.4	1.98	***p*** = 0.770
	Female	0	45	13.8	1.17	

The continuous variables are presented and analyzed via the Mann–Whitney *U*-test. Statistical significance is set at *p* < 0.05.

## 4 Discussion

There are several most important findings of this study. First, sex, tobacco consumption, and training load are found to be significantly associated with PDB among Swedish combat sport athletes. On the other hand, no significant association between doping knowledge and PDB was found. Therefore, our initial study hypothesis was not fully confirmed. Before discussing the mentioned results, we will briefly present the prevalence of PDB in studied athletes.

### 4.1 Prevalence

The prevalence of potential doping behavior in the present sample was 9.7%, indicating that approximately one in ten Swedish combat sport athletes demonstrated some degree of susceptibility to future doping behavior. However, this lower prevalence should be interpreted cautiously and not as direct evidence that Sweden is a lower-risk country for doping susceptibility. Previous research has shown that both prevalence and determinants of PDB vary across different sports and settings. Even under WADA anti-doping regulations, the implementation of anti-doping rules can differ between national sport systems in terms of education, testing, organizational structures, and cultural attitudes towards doping. Therefore, it is possible that country-specific prevention environments, including the Swedish sport system, and systematic anti-doping education employed by the Swedish Budo & Martial Arts Federation contribute to variations in PDB. However, the present cross-sectional study was not designed to test these mechanisms directly. Importantly, comparisons were limited to studies that employed the same standardized questionnaire and identical classification of PDB, allowing for more meaningful interpretation across different athletic populations. Compared with previous investigations using the same methodology, the prevalence observed in Swedish combat sport athletes was lower than that reported in high-level kickboxers (21.5%), rugby union players (23%), and competitive swimmers (16.3%), while remaining comparable to values previously reported in team-sport athletes (7, 8, 10, 15). Despite these between-sport differences, repeated observation of PDB across diverse athletic populations consistently demonstrates that susceptibility to doping is not restricted to a single sport or competitive setting. Direct comparisons should nevertheless be interpreted with caution because PDB reflects self-reported susceptibility rather than confirmed doping behavior, and may therefore be influenced by differences in sport-specific environments and social desirability bias (3, 14). Consequently, the present findings further support the need for sport- and country-specific anti-doping prevention strategies.

### 4.2 Males vs. females

The finding that female athletes demonstrated lower susceptibility to potential doping behavior than males was expected and is consistent with the study hypothesis. However, the observed sex difference in PDB should be interpreted with caution. Although male athletes showed a significant unadjusted association with higher PDB than females, no female participants were classified as high risk, and sex were therefore excluded in the multivariable model because of perfect separation. As a result, the present findings do not establish sex as an independent predictor of PDB, but rather indicate a sample-specific association that should be verified in larger studies with more accurate female representation. Moreover, this observation agrees with a substantial body of evidence indicating that male athletes are generally more prone to doping-related attitudes and behaviors across different sporting disciplines. Similar findings have been reported in studies involving swimmers and team sport athletes, suggesting that sex is one of the most consistently reported correlates of potential doping behavior (10, 15).

Several explanations can be offered to explain the lower susceptibility to doping among female athletes, including those involved in combat sports we have studied herein. Previous research suggests that women generally perceive doping as less acceptable and report stronger moral opposition to the use of prohibited substances than men (15, 27). Furthermore, females are generally known as being more cautious when evaluating behaviors associated with potential health-, ethical-, and career-related consequences (28). In the context of competitive sport, this naturally makes females less willing to engage in risky performance-enhancing practices, such as doping. Although these mechanisms are unlikely to fully explain the observed sex differences, they provide a plausible framework for explaining the presented differences between males and females in PDB. An additional explanation may relate to the competitive structure of combat sports. However, competitive pressure was not directly assessed in the present study, and therefore this interpretation should be considered speculative. Although male CS athletes may perceive stronger pressure to achieve performance advantages, this mechanism was not included in the present study and should be examined in future research using direct assessments of perceived competitive pressure and related psychological factors.

### 4.3 Substance misuse and PDB

The present findings showing a positive association between occasional tobacco use and PDB are consistent with a substantial body of evidence indicating that substance use and misuse are closely associated with doping vulnerability. Previous studies conducted using the same methodological approach have repeatedly reported that athletes engaging in behaviors such as tobacco use, alcohol misuse, or dietary supplement consumption exhibit greater susceptibility to potential doping behavior than their counterparts without such behaviors (7, 26). These findings are also supported by broader evidence suggesting that doping rarely occurs as an isolated behavior but rather represents part of a wider pattern of health-risk and performance-oriented behaviors (29). Consequently, substance use should not be viewed merely as an independent health concern but also as a potential marker for increased vulnerability to doping behavior. This association between occasional tobacco use and PDB should be interpreted with caution, as the wide confidence interval indicates limited precision. The association between occasional tobacco use and high PDB was statistically significant but imprecise, as the confidence interval ranged from 1.36 to 37.96. This limits strong causal inference, and the estimate should be considered suggestive rather than definitive. Given the small high-risk group, this trend should not be interpreted as evidence of a true dose-response relationship. One possible interpretation is that occasional tobacco use reflects broader substance-related vulnerability rather than nicotine exposure itself. In combat sports, nicotine may also be used for body-mass management, but remains a plausible explanation rather than a tested mechanism in the present study.

### 4.4 Training load and PDB

The positive association between training load and PDB was expected and is consistent with previous research conducted in other athletic populations. Although the present study is, to our knowledge, the first to examine accumulated training load with association to PDB, previous investigations using the same methodological approach have consistently shown that athletes exposed to more demanding training processes (i.e., higher training intensity, specific coaching strategies, and high-load training methodologies) demonstrate greater susceptibility to doping behavior (7, 10).

One possible explanation is rooted in the physiological purpose of many performance-enhancing substances. Historically, doping has primarily evolved as a means of accelerating recovery and enhancing adaptation to demanding training (30). Athletes exposed to higher training loads experience greater physiological stress and require more extensive recovery to achieve supercompensation (31). Consequently, when the training stimulus exceeds the athlete's natural recovery capacity, the perceived value of pharmacological strategies that facilitate recovery and adaptation may become substantially greater. In contrast, athletes exposed to lower training loads are more likely to achieve adequate recovery naturally and are therefore less prone to agents which could facilitate their recovery, including doping. Although not directly related to doping, studies that confirmed higher consumption of dietary supplements in advanced-level athletes indirectly support the previous discussion (32). Moreover, because of such a mechanism (greater stress due to higher training demands leads to dietary supplementation), usage of dietary supplements in the athletic population is often considered a certain “gateway to doping” (33, 34).

An additional explanation may relate to specific context of training load. Namely the training load may also be viewed as an indirect but consistent marker of the competitive environment in which athletes perform. Athletes of higher performance level generally train more, and higher training loads depict the increasing demands required to remain competitive at advanced levels (35). Consequently, athletes exposed to such environments may perceive stronger pressure to obtain even minimal advancement in the trained capacity. Logically, this competitive context may further contribute to the observed association between higher training load and more positive PDB. The discussed positive association between training load and PDB should be interpreted independently from the observed sex difference. Although male CS athletes showed higher susceptibility to PDB, training load did not differ significantly between male and female participants in the present sample. Therefore, greater training demands among males cannot be used to explain the sex difference observed. Instead, the finding suggest that higher training load was associated with PDB in the overall sample, while the mechanisms underlying the sex difference remain uncertain and should be examined in larger studies with more balanced sex representation.

### 4.5 Anti-doping knowledge and PDB

Contrary to our expectations, knowledge on doping was not associated with PDB. This finding is noteworthy because improving athletes' knowledge has long been considered one of the cornerstones of anti-doping prevention (9, 36). The idea is that the better-informed athletes will make more informed decisions and, consequently, be less susceptible to doping behavior (9). However, one possible explanation for the lack of association between exact knowledge on doping (evaluated by the questionnaire used in this study) and PDB lies in the multidimensional nature of anti-doping knowledge. In brief, the measurement tool used in this investigation consists of questions evaluating health-related risks and the hazard of doping use, and (ii) legal anti-doping literacy (12). Greater awareness of the health risks associated with doping (first construct of the measurement tool) may indeed discourage athletes from using doping by increasing their understanding of the adverse consequences. From this perspective, higher levels of knowledge would be expected to reduce susceptibility to doping behavior. At the same time, legal anti-doping literacy (second construct) encompasses regulatory aspects of the anti-doping system (i.e., familiarity with testing procedures, therapeutic use exemptions). Such knowledge may not necessarily discourage doping. Instead, it may simply reflect greater involvement in competitive sport or even an increased interest in understanding the anti-doping system among athletes (sometimes even in those athletes who perceive a higher likelihood of doping behavior). Consequently, the overall association between anti-doping knowledge and PDB may be altered because of the conceptually different forms of doping knowledge tested.

The heterogeneous anti-doping environment specific for combat sports observed in this study may provide an additional explanation for the lack of association between doping knowledge and PDB. It is well known that athletes from Olympic disciplines generally receive substantially more structured anti-doping education than those competing in many non-Olympic sports (9). As such, athletes competing in Olympic (combat) sports are routinely exposed to anti-doping education programs, testing procedures, and strict regulatory requirements, and such exposure is found to be a positive predictor of advanced knowledge on doping, especially anti-doping legal issues (9). Meanwhile, such exposure to anti-doping activities is considerably less consistent in many non-Olympic (combat) sports. Therefore, doping knowledge may reflect sport-specific regulatory practices rather than athletes' personal attitudes toward doping, which consequently reduces the association between knowledge on doping and PDB in combat sports. Although the self-reported factual doping knowledge was not significantly associated with PDB in this sample, a potentially valuable interpretation may be that factual knowledge alone might not be sufficient to discriminate doping susceptibility. However, the present study cannot directly test that explanation because values, attitudes, norms, self-regulatory efficacy, and several combat-sport-specific pressures were not measured directly. As a result, these interpretations should be seen as hypotheses for future research, rather than explanations supported by the present data.

### 4.6 Strengths and limitations

The study has several notable strengths. First, this is the first study to investigate PDB and its determinants in a large cohort of Swedish combat sport athletes (*n* = 165). Second, the sample included a range from non-competitors to internationally ranked medalists across multiple combat sport disciplines. Athletes were recruited nationwide through the official registry of the Swedish Budo & Combat Sports Federation. This broad recruitment strategy strengthens the geographic and competitive diversity of the sample. However, because the total number of athletes approached, the response rate, and the comparability of the sample to the broader Swedish combat sport population were not available. Hence, the representativeness of the findings should be interpreted with caution. Third, the use of standardized and previously validated questionnaires enhanced the reliability, validity, and comparability of the collected data with existing literature. Fourth, the study employed multivariable logistic regression analyses allowing adjustment for several potential confounders and identification of independent associations. Given the cross-sectional design, the heterogeneous sample, and the limited number of athletes classified as high risk for PDB (*n* = 16), these findings should be interpreted as exploratory evidence on correlates of PDB, rather than definitive determinants or clinical predictors.

However, several limitations should be acknowledged. A key limitation is the cross-sectional design, which precludes causal inferences regarding the relationships between the selected correlates and PDB. Because training load was derived from categorical weekly duration and weighted perceived-intensity frequencies rather than directly reported session-level RPE and duration, the units are indirect and its effect estimates should be interpreted cautiously. Second, the self-reported questionnaires used make the findings susceptible to recall bias, social desirability bias, and response bias, particularly given the sensitive nature of doping-related behaviors. Although anonymous data collection may have reduced these effects, some degree of underreporting or misreporting cannot be excluded. Third, the low representation of female compared with male participants limits the generalizability of the findings to female combat sport athletes and precludes robust sex-specific analyses. Fourth, the study assessed PDB rather than confirmed doping rule violations, and intentions or susceptibility may not necessarily translate into actual behavior. Moreover, unmeasured confounding factors, such as coaching environment, team or club culture, previous anti-doping education, psychological characteristics, and access to performance-enhancing substances, may have influenced the observed associations. Finally, since the sample consisted exclusively of Swedish combat sport athletes, the findings should be generalized cautiously to athletes from other countries, sporting cultures, or disciplines, where social norms, anti-doping policies, and competitive environments may differ. Although penalized methods such as Firth logistic regression may be useful in sparse-data settings, the present analysis was retained as an exploratory multivariable model. Therefore, the reported odds ratios should be interpreted cautiously given the limited number of high-PDB cases and the possibility of sparse-data bias.

## 5 Conclusions

The present study provides initial evidence on PDB and selected correlates among Swedish combat sport athletes. The findings suggest that athletes with greater training demands and those reporting sporadic tobacco use may be at increased risk of PDB, which can help inform hypotheses and future targeted prevention research. However, these results provide important baseline data for Swedish combat sports and contribute to a better understanding of factors associated with doping susceptibility. Future research should include larger and more diverse samples, particularly a greater representation of female athletes and competitors across different performance levels and employ longitudinal and intervention designs to evaluate the effectiveness of preventive strategies in reducing PDB and its underlying correlates and mechanisms. These findings should be interpreted as exploratory associations based on a limited number of high-PDB cases.

Future studies should examine whether some potentially important factors (i.e., injury-related recovery demands, nicotine use for body-mass control, competitive pressure, and sex-specific factors) contribute to PDB among combat sport athletes. Direct assessment of these mechanisms, preferably using longitudinal designs and larger, sex-balanced samples, would provide a clearer understanding of the observed associations.

## Data Availability

The raw data supporting the conclusions of this article will be made available by the authors, without undue reservation.

## References

[1] Damjanovic S Rossi C Manolopoulos N Matijevic T Korpak D Kurtanovic A. Doping in combat sports: a systematic review. Phys Sportsmed. (2025) 53(2):103–11. 10.1080/00913847.2025.2449812 39754586

[2] Hayward G Gaborini L Sims D Schumacher YO Millet GP Rhumorbarbe D. The athletic characteristics of Olympic sports to assist anti-doping strategies. Drug Test Anal. (2022) 14(9):1599–613. 10.1002/dta.3329 35732071

[3] WADA. (2025). Available online at: https://www.wada-ama.org/en/resources/anti-doping-stats/anti-doping-rule-violations-adrvs-report (Accessed February 22, 2026).

[4] Hurst P King A Massey K Kavussanu M Ring C. A national anti-doping education programme reduces doping susceptibility in British athletes. Psychol Sport Exerc. (2023) 69:102512. 10.1016/j.psychsport.2023.102512 37665917

[5] Kim Y An D. Doping susceptibility in Korean athletes: a validation study of the ADBS-K. J Sci Med Sport. (2026). 10.1016/j.jsams.2026.05.004 42168023

[6] Ntoumanis N Dølven S Barkoukis V Boardley ID Hvidemose JS Juhl CB. Psychosocial predictors of doping intentions and use in sport and exercise: a systematic review and meta-analysis. Br J Sports Med. (2024) 58(19):1145–56. 10.1136/bjsports-2023-107910 38925889 PMC11503166

[7] Sekulic D Bjelanovic L Pehar M Pelivan K Zenic N. Substance use and misuse and potential doping behaviour in rugby union players. Res Sports Med. (2014) 22(3):226–39. 10.1080/15438627.2014.915839 24950111

[8] Sekulic D Zenic N Versic S Maric D Gabrilo G Jelicic M. The prevalence and covariates of potential doping behavior in kickboxing; analysis among high-level athletes. J Hum Kinet. (2017) 59:67–77. 10.1515/hukin-2017-0148 29134049 PMC5680687

[9] Sinkovic A Pivalica D Jukic I Pehar M Pivalica B Cerkez Zovko I. The effect of educational intervention on legal anti-doping knowledge and doping tendency in elite athletes. Sports (Basel). (2026) 14(1):35. 10.3390/sports14010035 41590977 PMC12846038

[10] Liposek S Zenic N Saavedra JM Sekulic D Rodek J Marinsek M. Examination of factors explaining coaching strategy and training methodology as correlates of potential doping behavior in high-level swimming. J Sports Sci Med. (2018) 17(1):82–91. 29535581 PMC5844212

[11] Petróczi A Gleaves J De Hon O Sagoe D Saugy M. Prevalence of doping in sport. In: Mottram DR Chester N, editors. Drugs in Sport. New York: Routledge (2022). p. 37–71.

[12] Kondric M Sekulic D Uljevic O Gabrilo G Zvan M. Sport nutrition and doping in tennis: an analysis of athletes’ attitudes and knowledge. J Sports Sci Med. (2013) 12(2):290–7. 24149808 PMC3761838

[13] Rodek J Idrizovic K Zenic N Perasovic B Kondric M. Differential analysis of the doping behaviour templates in three types of sports. Coll Antropol. (2013) 37(Suppl 2):211–7. 23914509

[14] Rodek J Sekulic D Kondric M. Dietary supplementation and doping-related factors in high-level sailing. J Int Soc Sports Nutr. (2012) 9(1):51. 10.1186/1550-2783-9-51 23217197 PMC3536606

[15] Sekulic D Tahiraj E Zvan M Zenic N Uljevic O Lesnik B. Doping attitudes and covariates of potential doping behaviour in high-level team-sport athletes; gender specific analysis. J Sports Sci Med. (2016) 15(4):606–15. 27928206 PMC5131214

[16] Garcia SM Tor A. The N-effect: more competitors, less competition. Psychol Sci. (2009) 20(7):871–7. 10.1111/j.1467-9280.2009.02385.x 19538434

[17] Garcia SM Tor A Schiff TM. The psychology of competition: a social comparison perspective: a social comparison perspective. Perspect Psychol Sci. (2013) 8(6):634–50. 10.1177/1745691613504114 26173228

[18] Kusnierz C Niewczas M Cynarski WJ Bielec G Rogowska AM. Latent class analysis of aggression in martial arts and combat sports: a cross-sectional study. PLoS One. (2025) 20(7):e0328799. 10.1371/journal.pone.0328799 40700371 PMC12286348

[19] Lakicevic N Reale R D'Antona G Kondo E Sagayama H Bianco A. Disturbing weight cutting behaviors in young combat sports athletes: a cause for concern. Front Nutr. (2022) 9:842262. 10.3389/fnut.2022.842262 35223962 PMC8863958

[20] Artioli GG Saunders B Iglesias RT Franchini E. It is time to ban rapid weight loss from combat sports. Sports Med. (2016) 46(11):1579–84. 10.1007/s40279-016-0541-x 27102173

[21] Petrou M Lazuras L Hillier M Mojtahedi D. Doping behaviour in mixed martial arts athletes: the roles of social norms and self-regulatory efficacy. Int J Sport Exerc Psychol. (2022) 20(4):1086–101. 10.1080/1612197X.2021.1948587

[22] Wagner U Hanstad DV. Scandinavian perspectives on doping – a comparative policy analysis in relation to the international process of institutionalizing anti-doping. Int J Sport Policy Politics. (2011) 3:355–72. 10.1080/19406940.2011.596156

[23] Kvillemo P Gripenberg J Strandberg AK Elgán TH. Police officers’ perspective on doping and prevention among recreational athletes: a cross-sectional study. Front Sports Act Living. (2023) 5:1251531. 10.3389/fspor.2023.1251531 37936878 PMC10626526

[24] Andreasson J Henning A. Glocal fitness doping: policy, practice and prevention in the United States and Sweden. Perform Enhanc Health. (2019) 6(3–4):103–10. 10.1016/j.peh.2018.11.001

[25] Cuschieri S. The STROBE guidelines. Saudi J Anaesth. (2019) 13(Suppl 1):S31–4. 10.4103/SJA.SJA_543_18 30930717 PMC6398292

[26] Kondric M Sekulic D Petroczi A Ostojic L Rodek J Ostojic Z. Is there a danger for myopia in anti-doping education? Comparative analysis of substance use and misuse in Olympic racket sports calls for a broader approach. Subst Abuse Treat Prev Policy. (2011) 6:27. 10.1186/1747-597X-6-27 21988896 PMC3204239

[27] Bloodworth A Petróczi A Bailey R Pearce G McNamee M. Doping and supplementation: the attitudes of talented young athletes. Scand J Med Sci Sports. (2012) 22(2):293–301. 10.1111/j.1600-0838.2010.01239.x 20973831

[28] Byrnes JP Miller DC Schafer WD. Gender differences in risk taking: a meta-analysis. Psychol Bull. (1999) 125(3):367–83. 10.1037/0033-2909.125.3.367

[29] Blank C Kopp M Niedermeier M Schnitzer M Schobersberger W. Predictors of doping intentions, susceptibility, and behaviour of elite athletes: a meta-analytic review. SpringerPlus. (2016) 5(1):1333. 10.1186/s40064-016-3000-0 27563528 PMC4980857

[30] Gleaves J Hunt T. A Global History of Doping in Sport: Drugs, Policy, and Politics. London: Taylor & Francis (2016).

[31] Plowman S Smith D. Exercise Physiology for Health, Fitness, and Performance. Philadelphia: Lippincott Williams & Wilkins (2007).

[32] Erdman KA Fung TS Reimer RA. Influence of performance level on dietary supplementation in elite Canadian athletes. Med Sci Sports Exerc. (2006) 38(2):349–56. 10.1249/01.mss.0000187332.92169.e0 16531906

[33] Hurst P. Are dietary supplements a gateway to doping? A retrospective survey of athletes’ substance use. Subst Use Misuse. (2023) 58(3):365–70. 10.1080/10826084.2022.2161320 36645808

[34] Mallick M Camacho CB Daher J El Khoury D. Dietary supplements: a gateway to doping? Nutrients. (2023) 15(4):881. 10.3390/nu15040881 36839238 PMC9964767

[35] Bompa TO Buzzichelli C. Periodization-6th Edition: Theory and Methodology of Training. Champaign: Human Kinetics (2019).

[36] Houlihan B McNamee M. Anti-doping: Policy and Governance. London: Taylor & Francis (2014).

